# Structure-tuned Ti_3_C_2_T_*x*_ MXene for efficient adsorption and visible-light-assisted removal of dye and pharmaceutical contaminants

**DOI:** 10.1039/d6ra04015b

**Published:** 2026-07-03

**Authors:** Bahram Ahmadi, Masoud Jamshidi, Reza Ghamarpoor

**Affiliations:** a Constructional Polymers and Composites Research Lab., School of Chemical, Petroleum and Gas Engineering, Iran University of Science and Technology (IUST) Tehran Iran mjamshidi@iust.ac.ir; b Department of Petroleum Engineering, Faculty of Engineering, University of Garmsar Garmsar Iran rezaghamarpoor@fmgarmsar.ac.ir

## Abstract

Finding effective materials for the removal of pollutants from wastewater has become an essential requirement in environmental protection and the development of advanced water-treatment technologies. In this study, Ti_3_C_2_T_*x*_-MXene was synthesized using two methods (*i.e.*, sol–gel and hydrothermal). The influence of synthesis parameters, including etching agent (*i.e.*, LiF and NaF), temperature (*i.e.* 38, 80 and 90 °C), and reaction time (*i.e.*, 3 and 7 days), on the structural characteristics and pollutant-removal performance of MXene was systematically investigated. The synthesized samples were evaluated for the removal of dye and pharmaceutical contaminants, including methylene blue (MB), tetracycline (TC), carbamazepine (CBZ), and phenazopyridine (PhP). The results revealed that the sample synthesized using LiF (*i.e.*, LTi2) as an etchant at 90 °C possessed the highest specific surface area and exhibited higher adsorption efficiency compared to other samples. At an initial concentration of 10 ppm, LTi2 removed more than 93% of MB and 88% of PhP within 60 min through adsorption. At a concentration of 40 ppm, adsorption efficiencies of approximately 48%, 29%, and 18% were obtained for MB, TC, and CBZ, respectively. Under subsequent visible-light irradiation, further pollutant removal was observed, resulting in total removal efficiencies exceeding 98% for MB and 93% for PhP at 10 ppm, while overall removal efficiencies of 59%, 64%, and 37% were achieved for MB, TC, and CBZ, respectively, at 40 ppm. These findings demonstrated that careful control of the synthesis conditions was an effective strategy for tailoring the MXene structure and improving its adsorption-dominated and visible light assisted pollutant removal performance.

## Abstract 2

Finding effective materials for the removal of pollutants from wastewater has become an essential requirement in environmental protection and the development of advanced water-treatment technologies.

## Graphical abstract

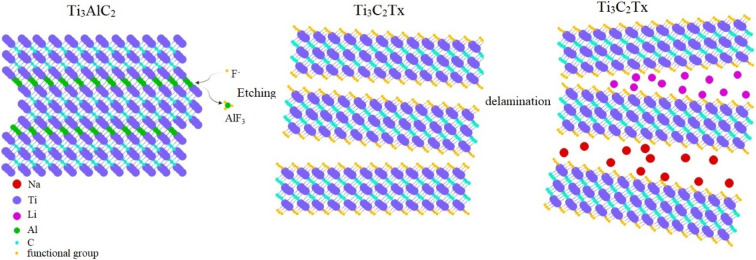

## 1 Introduction

MXenes represent a family of two-dimensional materials composed of transition-metal carbides, nitrides, or carbonitrides, exhibiting layered structures with tunable chemical compositions.^1–4^ MXenes have emerged as one of the most extensive and rapidly evolving classes of two-dimensional materials, drawing substantial interest across research and industrial sectors.^5^ The multilayer atomic structure of MXene formed after the etching process exhibits a sandwich-like architecture, in which *n* + 1 layers of M atoms are alternately arranged with *n* layers of X atoms, while surface functional groups (T) are attached to the M atoms on the outer layers.^6,7^ The formation of these open, accordion-like structures is attributed to the liberation of hydrogen gas (H_2_) resulting from the reaction between aluminum (Al) in the MAX phase and hydrofluoric acid (HF). Within the MAX framework, the M–X bonds are characterized by a mix of ionic, metallic, and covalent interactions, while the M–A bond exclusively exhibits metallic properties.^8–10^

As a consequence of their vast electrochemically active surface area, remarkable electrocatalytic properties, and high electrical conductivity, these materials have been used in a wide range of applications.^11–13^ The unique combination of the hydrophilicity of graphene oxide with the superior electrical conductivity characteristic of metals in the MXene structure has paved the way for innovative applications in areas such as energy storage, electromagnetic interference shielding, actuator systems, and communications.^5,14,15^ The wide diversity in the selection of M and X elements, coupled with the value of *n*, enables the targeted tuning of the structural and surface chemical properties of these materials. Therefore, MXenes are recognized as a distinct and broader family compared with other two-dimensional materials.^16,17^

The production of MXenes is typically achieved by the preferential stripping of the A element from the MAX phase during the etching procedure. This step directly determines the layered structure, surface chemistry, and the inherent functional properties of the material. The basis of this process originates from the differing bond energy values between the M–A and M–X bonds; since the M–A bond is weaker, it can be separated more easily. When temperature is used as the etching agent, both types of bonds are broken, thereby leading to the creation of a three-dimensional structure. Among etching agents, compounds such as HF, LiF, and HCl are most commonly used.^18–20^

In MAX phases containing aluminum, the intensity of the etching process depends on the atomic number of the transition metal. As this number increases, the M–Al bond becomes stronger and requires harsher etching conditions. This process leads to the formation of active surface functional groups that enhance the interaction between MXene layers and polymer chains, improve adhesion, regulate interlayer spacing, and create more stable composites.^21,22^

Operational factors including the type of etchant, concentration, temperature, and time significantly influence the physical and chemical properties of the final product. For example, increasing the HF concentration leads to the formation of smaller flakes with a greater number of fluorinated groups, whereas the use of an HF–HCl mixture produces larger sheets with fewer defects. Additionally, increasing the pH of the solution converts OH groups into O groups, and increasing the weight percentage of HF increases surface roughness.^23,24^

Moreover, the conditions of the etching reaction, including temperature, reaction duration, and the type of fluoride salt also have a considerable influence on the final quality of MXene nanosheets. In general, increasing the temperature accelerates the reaction rate and enhances the effective removal of the Al layer. However, it also increases the likelihood of oxidation, structural degradation, and reduction in flake size. In contrast, lower temperatures help preserve structural integrity and produce sheets with fewer defects, although the etching process proceeds more slowly under these conditions. Additionally, choosing between LiF and NaF can lead to differences in surface functional groups and interlayer behavior, thereby influencing the dispersion, electrical conductivity, and stability of the nanosheets.^25,26^

To this end, numerous studies have been conducted in this field. For instance, Rao *et al.*^27^ reported that both the type of fluoride salt and the etching temperature significantly influence the Al extraction efficiency and the MXene yield, with NH_4_F exhibiting superior performance under optimal conditions. In another study, Liew *et al.*^28^ demonstrated that increasing the synthesis temperature markedly alters the morphology, elemental composition, and specific surface area of Ti_3_C_2_T_*x*_. Investigations by Lokhande *et al.*^29^ and Rajavel *et al.*^30^ also indicated that prolonged etching leads to aluminum removal and interlayer expansion; however, excessively harsh conditions tend to induce structural defects and consequently degrade the material's properties. In a study conducted by Yadav *et al.*^31^ it was shown that reaction temperature and time are key factors governing exfoliation and surface functionalization. Furthermore, Abdullah *et al.*^32^ and Yaman *et al.*^33^ emphasized that the choice of fluoride-containing etchants strongly affects the interlayer spacing, surface terminations, impurity content, and overall structural integrity of the resulting MXene.

Consequently, previous studies indicated that the type and concentration of etching agent, temperature, reaction time, and synthesis environment are fundamental factors in effective Al layer removal and Ti_3_C_2_T_*x*_ structural stability. Using mild and controlled conditions, particularly in LiF/HCl or NaF/HCl systems, leads to formation of uniform structures and as well as robust functional groups like as O^−^, OH^−^, and F^−^, while high temperature or high etching agent concentration results in structural defects and reduced electrical conductivity. Over the past several years, the use of alkaline environments and phosphate compounds or molten salts as green pathways has improved layer ordering and enhanced electrochemical performance. On the other hand, investigation of the role of various metal ions has shown that ion type significantly affects the Al removal process, surface functional group density, and interlayer spacing. Specifically, due to its larger ionic size, NaF results in milder etching and the formation of more ordered structures, while also inducing changes in the surface chemical composition.^16,34,35^

However, despite all the progress made in the synthesis of MXenes, the combined effect of the type of fluoride salt, etching temperature, and reaction time on their structural properties particularly the specific surface area, and their pollutant removal performance has not yet been sufficiently understood. Therefore, the aim of this work is to establish a direct relationship between synthesis conditions, material properties, and pollutant removal behavior.^36,37^

In recent years, researchers have used two-dimensional materials, particularly MXenes, for the adsorption of pollutants.^38^ Tran *et al.*^39^ reported that Ti_3_C_2_T_*x*_ MXene can effectively adsorb MB from wastewater. The material was synthesized by HF etching of the Ti_3_AlC_2_ MAX phase and was mainly terminated with F functional groups. Their results showed that about 92% of methylene blue in a 20 µM aqueous solution was removed within 5 minutes. They also found that the adsorption efficiency strongly depends on a function of solution pH and the MXene's surface functionalization. Furthermore, the MXene exhibited good recyclability, highlighting its applicability in real-world wastewater remediation.

To address this, the present investigation was undertaken to examine how different types of fluoride ions impact the exfoliation process, specific surface area, and structural modification of Ti_3_C_2_ MXene, NaF and LiF were used in acidic HCl medium. The initial Ti_3_AlC_2_ phase was synthesized by two different methods at temperatures of 38 and 80 °C over etching times of 3 and 7 days to evaluate the effect of influential parameters including etching agent type, temperature, and reaction duration on the structural characteristics, morphology, specific surface area, functional groups, and physical properties of the final product. Furthermore, the synthesis, washing, delamination, and drying methods are explained step-by-step and in detail, which has been addressed in few articles. Finally, the samples were subjected to adsorption and photocatalytic tests with various pharmaceutical pollutants and the reference dye MB to determine the effect of these parameters on pollutant adsorption and degradation. Additionally, to prevent secondary pollution formation, the optimized sample was incorporated into an acrylic polymer film for stable utilization. The results of this investigation, aimed at determining optimal conditions for producing Ti_3_C_2_T_*x*_ nanosheets with ordered structure, appropriate stability, and environmental compatibility for application in the field of pollutant adsorption, represent a strategic advancement in the environmental domain for protecting a clean Earth.

## 2 Experimental section

### 2.1 Materials

The materials used in this study are precisely listed in Table 1.

**Table 1:** Chemicals used in this study, abbreviation name, and manufacturer company

No	Materials	Abbreviation	Source
1	Ti_3_AlC_2_	Ti_3_AlC_2_	Alfa Aesar
2	HCl	HCl	Dr Mojallali Co. (Iran)
3	Lithium fluoride	LiF	Sigma-Aldrich Co.
4	Sodium fluoride	NaF	Sigma-Aldrich Co.
5	Ethanol	Eth	Dr Mojallali Co. (Iran)
6	Methylene blue	MB	Dr Mojallali Co. (Iran)
7	Tetracycline 250 mg	TC	Iran Daru Co. (Iran)
8	Carbamazepine 400 mg	CBZ	Actover Co. (Iran)
9	Phenazopyridine 100 mg	PhP	Iran Daru Co. (Iran)
10	Acrylic latex R-84	AcL	Simab Resin Co. (Iran)

### 2.2 Construction of Ti_3_C_2_T_*x*_

MXene synthesis was performed using different methods (See Table 2).

**Table 2:** Different synthesis pathways

Codes	Etching agent	Solvent	MAX-phase	Synthesis method	Days	Temperatures (°C)
LTi1	—	—	—	—	—	38 °C
LTi2	LiF (0.35 g)	HCl, (20 ml)	Ti_3_AlC_2_, (0.3 g)	Sol–gel	3	90 °C
NTi1	NaF (0.35 g)			Hydrothermal	3	80 °C
NTi2					7	

#### 2.2.1 First method

0.35 g of LiF was added to 18 cc of HCl and placed in a Teflon vessel on a stirrer for 10 min at 650 rpm until the LiF particles were completely dissolved. Subsequently, 0.3 g of Ti_3_AlC_2_ was slowly added over 2 min to prevent exothermic reactions. After complete addition of the MAX phase, the vessel was sealed and placed in a water bath for 3 days at temperatures of 38 °C and 90 °C.

#### 2.2.2 Second method

0.35 g of NaF was added to 18 cc of HCl acid in a Teflon vessel and placed on a stirrer with the lid closed for 20 min until the NaF was completely dissolved. Then, 10 cc of deionized water was slowly added to the solution to adjust the etchant concentration. In the final step, the clear solution was transferred to a hydrothermal vessel and the MAX phase was slowly added. The vessel was sealed and maintained at 80 °C for 3 and 7 days.

The washing procedures were performed identically for all synthesis methods. After completion of the etching process, the resulting solution was collected by centrifugation at 5000 rpm for 5 min. In the first step, the sample was washed twice with HCl solution to remove residual Al compounds. Subsequently, five washes with ethanol were performed to remove organic compounds, unstable fluoride ions, and reduce the surface tension of particles, which resulted in better dispersion and prevention of MXene sheet aggregation. The samples were then washed five times with an equimolar solution of ethanol and water to allow gradual solvent exchange and prevent structural shock to the layers. Finally, to completely remove Li^+^, and F^−^ ions, washing with deionized water was repeated 25 time until complete clarification of the supernatant. This step was essential for achieving neutral pH and ensuring removal of all corrosive residues. Strict adherence to these steps resulted in obtaining Ti_3_C_2_T_*x*_ nanosheets with high purity, appropriate chemical stability, and uniform layered structure.

In the subsequent stage, ultrasonication was performed to separate the nanolayers and increase the interlayer spacing. This step plays a key role in breaking hydrogen and van der Waals bonds between Ti_3_C_2_T_*x*_ sheets and promotes more uniform dispersion of the flakes in aqueous medium. For this purpose, the sample was dispersed in deionized water and placed in a two-neck flask to simulate inert nitrogen gas conditions. This measure was necessary to prevent surface oxidation of MXenes in contact with oxygen and moisture, as Ti_3_C_2_T_*x*_ layers in aqueous environments, especially in the presence of dissolved ions and oxygen, rapidly undergo oxidation to TiO_2_. The ultrasonication process was performed at 70 watts for 10 min to effectively achieve layer separation without damaging the crystalline structure. After completion of the operation, the solution was centrifuged again and washed once with ethanol. The sample was then dispersed in ethanol and dried in a vacuum oven at 80 °C for 4 h. This vacuum drying step resulted in complete solvent evaporation and prevention of thermal oxidation at high temperatures, ultimately yielding Ti_3_C_2_T_*x*_ nanosheets with stable layered structure and uniform dispersion.

### 2.3 Photocatalytic test

Photocatalytic testing of the samples was performed under visible light irradiation. Prior to initiating the photocatalysis process, the suspension containing photocatalyst and pollutant was stirred for one hour in darkness to establish adsorption–desorption equilibrium and separate the contribution of physical adsorption from the photodegradation process. The pollutants were prepared at a concentration of 10 ppm. The pharmaceutical pollutants included tetracycline, carbamazepine and phenazopyridine. MB was also used as a dye pollutant. After the dark period, the samples were exposed to visible light irradiation for 6 hours in a light chamber with 32 watts power. During the process, sampling was performed at one-hour intervals and changes in pollutant concentration were examined through optical absorption measurements.

In all photocatalytic experiments, the notation “*d*” represents the initial 1 h dark adsorption period used to establish adsorption–desorption equilibrium prior to visible-light irradiation, whereas the labels 1–6 indicate irradiation time in hours.

### 2.4 Fabrication of the AcL polymer film

The optimal sample was selected at 2 wt% relative to resin mass and placed in 20 cc of water under ultrasonic bath for 30 minutes. It was then mixed with 50 g of AcL for 1 hour at 800 rpm to ensure uniform distribution. After mixing, this mixture was left to rest for 24 hours to allow bubbles generated during the preparation process to escape from the medium. Finally, the coating film was applied using a film applicator on a glass substrate such that the layer thickness was approximately 400 µm, and the samples were kept in dark conditions for 24 hours to complete the drying and curing process of the film.^40^

### 2.5 Characterization and test

To investigate the effect of etchant type, temperature, and reaction time, structural and microscopic analyses were performed to determine the crystal structure, morphology, and specific surface area of these nanoparticles. In this regard, the crystal structure and phase purity of the nanoparticles were examined using an X-ray diffraction (XRD) instrument (Bourevestnik, DRON-8, manufactured in Russia). Chemical bonding patterns, molecular functional groups, and chemical bonds were analyzed through Fourier-transform infrared spectroscopy (FT-IR) using a Spectrum 100 model (PerkinElmer USA). Surface morphology, layered structure, and molecular element distribution were investigated by field emission scanning electron microscopy (FESEM) and energy-dispersive X-ray spectroscopy (EDS) (MIRA3, TESCAN, Czech Republic). The specific surface area and porosity of the samples were examined through nitrogen adsorption–desorption isotherms using the BET method with a Belsorp mini II instrument (MicrotracBEL, Japan).

## 3 Results and discusstion

### 3.1 Structure of nanosheets

**Fig. 1:**
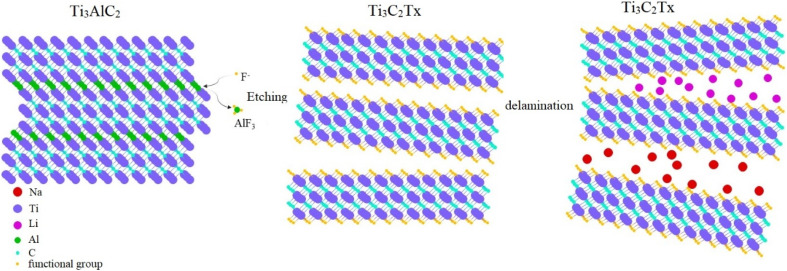
Schematic illustration of the synthesis of MXene nanosheets from the MAX phase.

A schematic representation of the Al layer removal process and the formation of bilayer MXene structures is illustrated in Fig. 1. A precise understanding of this process determines the main pathways for adjusting surface groups, sheet structure, and surface roughness. The initial Ti_3_AlC_2_ structure consists of alternating Ti–C layers and intermediate Al layers. According to the chemical reaction below, this structure undergoes selective etching in the presence of fluoride ions (F^−^) derived from fluoride compounds such as LiF or NaF in an acidic medium, and its Al layer is removed from the structure. Subsequently, the multilayer Ti_3_C_2_T_*x*_ structure, which is covered by functional groups (T_*x*_ = O^−^, OH^−^, F^−^), was subjected to sonication so that the presence of interlayer Li^+^ or Na^+^ ions could reduce the interlayer energy and form multilayer MXene nanosheets.

Gravimetric analysis of the dried MXene powder obtained after synthesis revealed that the yield of Ti_3_C_2_T_*x*_ was strongly dependent on the type of fluoride salt, etching temperature, and reaction duration. The highest yield was obtained for the sample synthesized with the HCl/LiF system at 38 °C, reaching 73.3%. This high yield can be attributed to the effective role of Li^+^ ions in interlayer penetration of the MAX phase and facilitation of the delamination process, and it is likely that the small ionic radius of Li^+^ provided easier access to the interlayer space of Ti_3_C_2_T_*x*_, resulting in more effective removal of the Al phase and formation of the MXene structure with minimal side degradation. Furthermore, conducting the process at the relatively low temperature of 38 °C reduced undesired side reactions, including Ti oxidation and edge degradation of the sheets, and contributed to preserving the final product mass.^41^

In contrast, increasing the process temperature in the HCl/LiF system to 90 °C led to a decrease in synthesis yield to 69.6%. Although higher temperature increased the etching rate and Al removal, the intensification of reactions under these conditions increased the probability of layered structure degradation, formation of surface defects, and even partial dissolution of MXene sheets.^42^

In samples synthesized with the HCl/NaF system, the synthesis yield was noticeably lower than that of samples containing LiF. Specifically, the yield of the sample etched for 3 days was 50%, and for the sample etched for 7 days, it was 43.3%. This yield reduction is also likely related to the larger ionic radius of Na^+^, which limited its penetration into the interlayer space of the MAX phase, resulting in a more incomplete delamination process.

#### 3.1.1 XRD

**Fig. 2:**
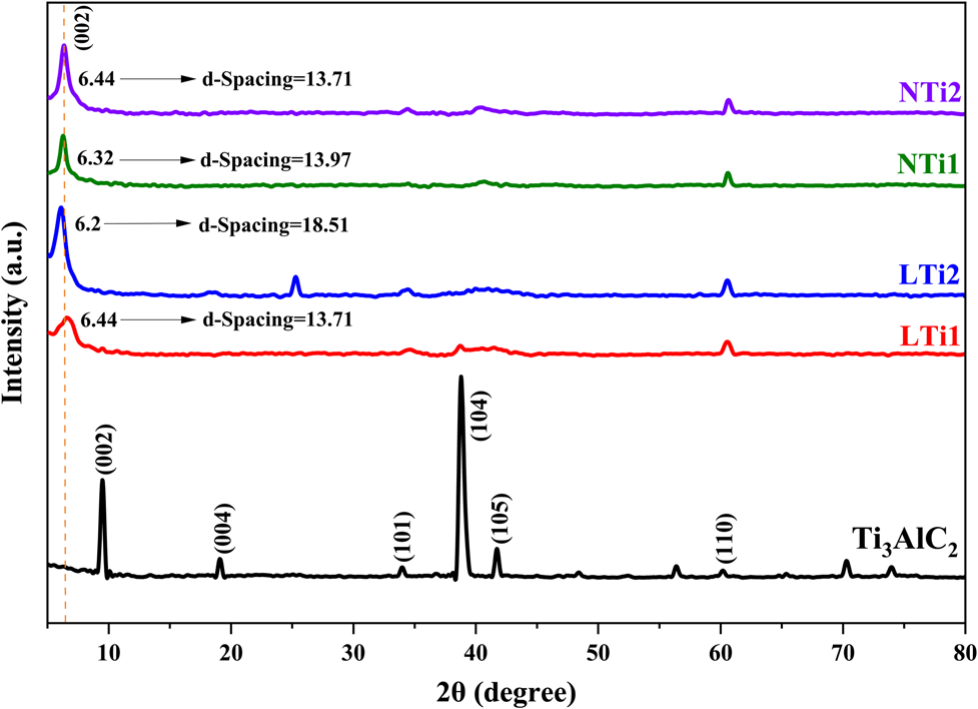
XRD patterns and corresponding crystalline phases of the MAX-phase and Ti_3_C_2_T_*x*_-MXenes.

XRD analysis was employed as a comprehensive method to evaluate successful synthesis and the increase in interlayer spacing. The disappearance of the (104) peak at 2*θ* = 38°, which corresponds to aluminum, and the shift of the (002) peak from 9.8° to lower angles, indicating an increase in the interlayer spacing of the sheets, were the most important XRD peaks related to MXene nanosheets, providing evidence for successful synthesis. As shown in Fig. 2, the characteristic peaks of Ti_3_AlC_2_ appeared at 2*θ* angles of approximately 9.5°, 19°, 34°, 36°, 38°, and 60°, corresponding to the (002), (004), (101), (104), (105), and (110) planes. The presence of these peaks confirmed the layered crystalline order in the structure of this material and the absence of impurities in the initial sample. After performing the etching process with different compositions, significant changes in the intensity and position of the peaks were observed. In samples etched with HCl/LiF solution (*i.e.*, LTi1 and LTi2), changes in the (002) peak from an angle of approximately 9.8° to the region of 6.2°–6.44° were observed. This shift toward lower angles indicated an increase in the interplanar spacing from approximately 9.4 Å in the MAX phase to values of 13.75 Å. Such an increase in spacing represents the opening of layers and the intercalation of lithium ions and water molecules during the etching process. Furthermore, the significant reduction in the intensity of the (104) and (105) peaks and their fading confirmed that the Al layer was almost completely removed from the structure. Additionally, the interplanar spacing value for the sample synthesized at 38 °C was obtained as 13.71 Å. Increasing the temperature from 38 °C to 90 °C in HCl/LiF solution exerted its effect on the position and intensity of the (002) peak, causing this peak to shift from 6.44° to 6.22° and increasing its intensity. This is likely related to improved crystal quality and reduced structural defects due to more favorable thermal conditions. However, the higher temperature likely caused surface roughening, partial oxidation, and the formation of a small amount of TiO_2_ on the surface.^43^

It was predicted that this partial oxidation could enhance the photocatalytic degradation capability of MXene. Regarding samples etched with the HCl/NaF system (*i.e.*, NTi1 and NTi2) under hydrothermal conditions at 80 °C, more significant changes in the position of the (002) peak were observed. In the NTi1 sample, this peak appeared at 6.32° (*d* ≈ 13.97 Å), exhibiting greater interlayer swelling compared to the LiF/38 samples. The presence of Na^+^, due to its larger ionic radius compared to Li^+^, resulted in a greater increase in interplanar spacing and better penetration of water molecules into the structure. The NTi2 sample showed this peak at 6.44°, and *d* decreased to approximately 13.71 Å; this minor change is likely related to relative compaction of the layers or partial removal of interlayer water during the longer hydrothermal etching duration.

#### 3.1.2 FE-SEM

**Fig. 3:**
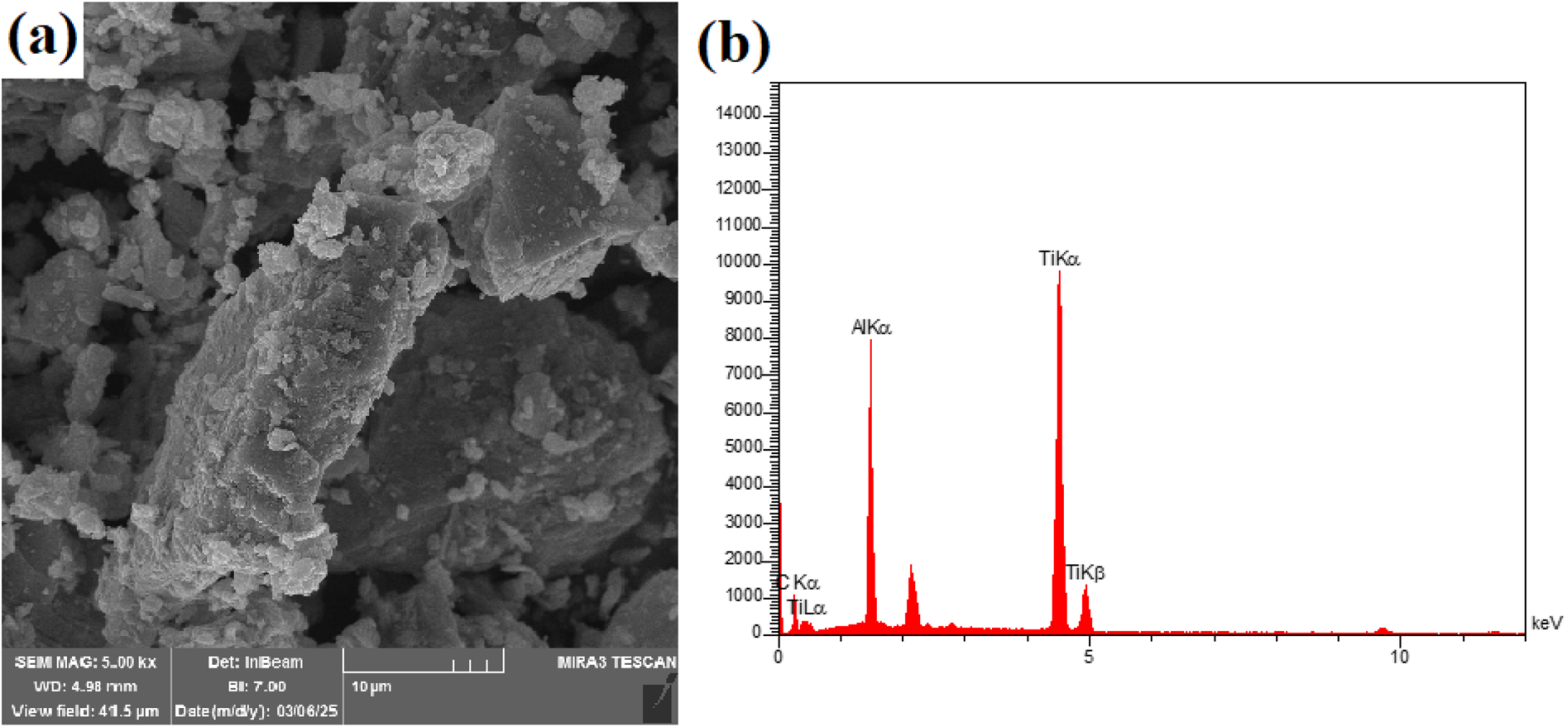
(a) FESEM images and (b) EDS elemental analysis of the MAX-phase.

FESEM images of the MAX-phase are presented in Fig. 3. A layered and compact structure of plates was observed, indicating the ceramic-like and metal-like nature of these materials. The presence of strong peaks of Ti and Al elements confirmed that the sample was enriched with these elements and was consistent with the composition of the MAX phase such as Ti_3_AlC_2_.

**Fig. 4:**
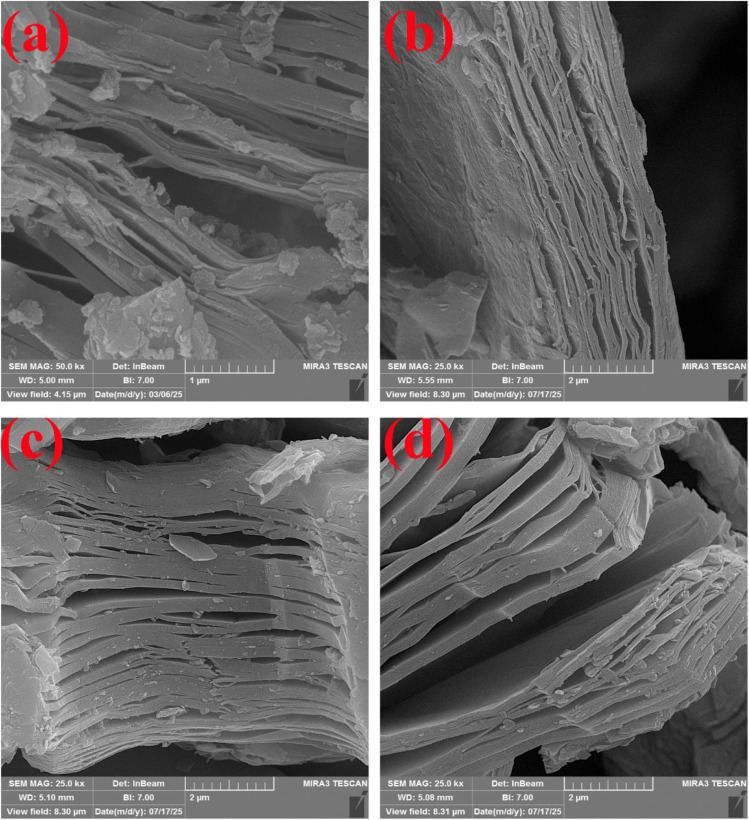
FE-SEM images of MXenes synthesized by different methods: (a) LTi1, (b) LTi2, (c) NTi1, and (d) NTi2.

Fig. 4a–d shows the morphology of Ti_3_C_2_T_*x*_ samples synthesized by four different methods. In all images, a layered and sheet-like structure was observed, but there were significant differences in the degree of separation and interlayer swelling between different samples, which showed good agreement with the XRD data. In Fig. 4a, which corresponds to the LTi1 sample, a relatively compact structure was seen, where the layers were very close to each other and had not completely separated. From this morphology and XRD analysis, it can be inferred that at lower temperature, the etching process was performed incompletely and Li^+^ ions had limited penetration between the layers. However, increasing the temperature had a direct impact on the etching process; such that in Fig. 4b, which corresponds to the LTi2 sample, more regular, fewer-layered, and relatively more open layers were observed, which was consistent with the XRD results. However, the outer surface of the sample exhibited considerable roughness compared to other samples, which is probably due to the higher temperature compared to the LTi1 sample.

**Fig. 5:**
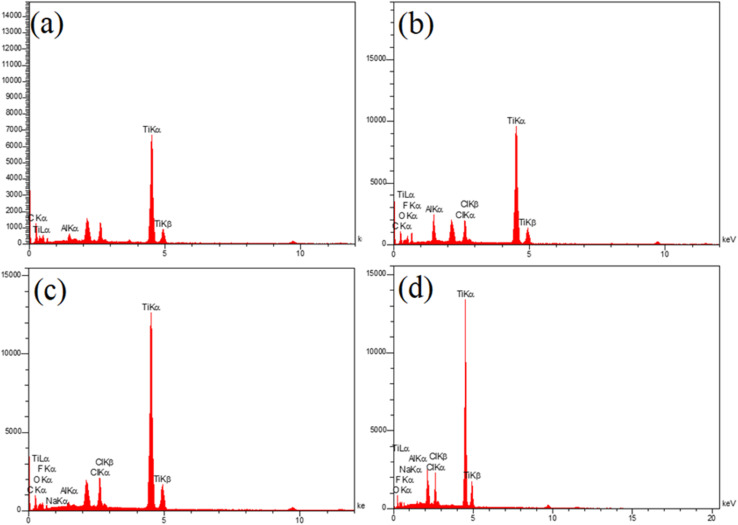
EDS elemental analysis of MXene synthesized using different methods: (a) LTi1, (b) LTi2, (c) NTi1, and (d) NTi2.

Replacing the etchant LiF with NaF and hydrothermal conditions created the morphology present in Fig. 4c and d. As Fig. 4c shows, the NTi1 sample possessed completely open layers with large spacings but had more sheets compared to the samples synthesized with LiF. Moreover, this structure had smooth and even surfaces, which indicates high success in the etching process and intercalation of Na^+^ ions. It can be assumed that these characteristics arose due to the larger ionic radius of Na^+^ compared to Li^+^ and hydrothermal conditions, leading to greater penetration and higher swelling of the accordion-like structure. In Fig. 4d, where the NTi2 sample is presented, the sheet-like structure was still maintained, but to some extent, re-compaction between the layers increased compared to other samples. This planar arrangement is probably due to the rearrangement of sheets during the longer time period and relative removal of interlayer water.^44,45^ In XRD, it was also observed that the position of the (002) peak shifted slightly to a higher angle (6.44°) and *d* decreased to 13.71 Å, which corresponds to the microscopic image of this sample. Furthermore, elemental analysis of these samples indicated relatively complete removal of Al in the structure, which is summarized in Fig. 3b, 5, and Table 3.

**Table 3:** EDS analysis results of the different synthesized samples

Sample codes	Elemental						
	Ti	Al	C	Na	O	F	Cl
Ti_3_AlC_2_-MAX	52.44	34.03	13.54	0	0	0	0
LTi1	49.56	6.31	10.21	0	14.25	8.43	11.24
LTi2	51.42	10.33	8.95	0	9.75	11.97	7.58
NTi1	67.05	2.34	6.82	1.52	8.74	5.81	7.72
NTi2	69.09	1.74	5.57	1.61	8.18	5.76	8.05

#### 3.1.3 FTIR

**Fig. 6:**
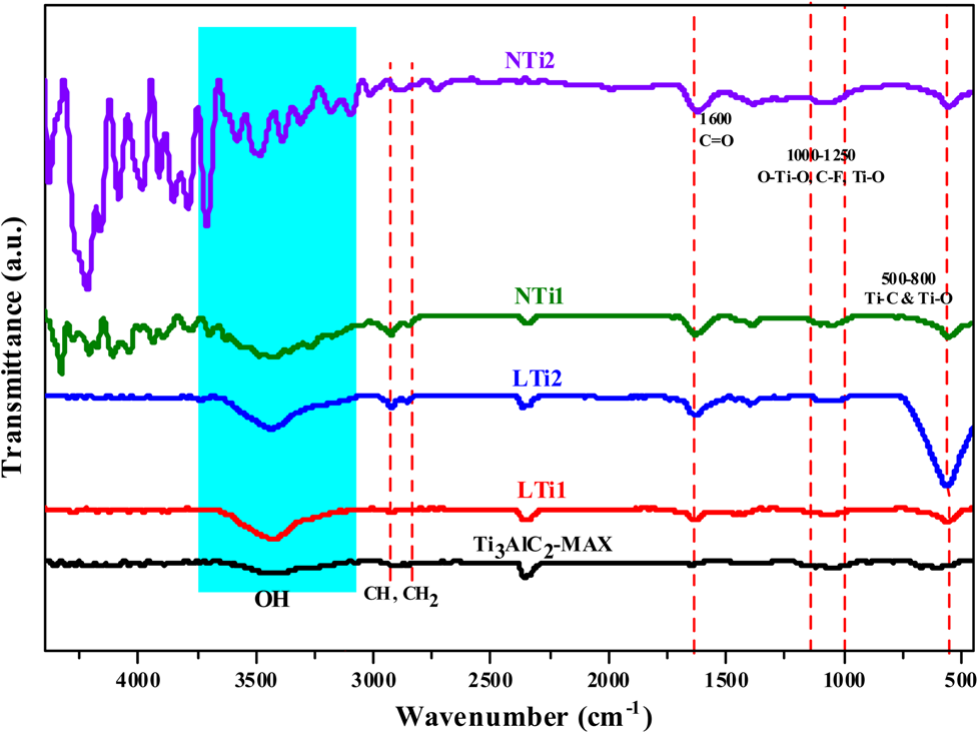
FTIR spectra of the MAX phase and MXene synthesized using different methods.

FTIR analysis was performed to investigate the surface chemistry alterations and the types of functional groups formed in the synthesized MXenes under different etching conditions. As shown in Fig. 6, comparison of the spectra revealed that after the etching process, new bands appeared in all MXene samples that were either very weak or not observed in the pristine MAX phase, indicating the removal of the Al phase and modification of the Ti_3_C_2_T_*x*_ surface chemistry. One of the most characteristic changes is the broad and relatively intense band in the region around 3012–3403 cm^−1^, which was attributed to the stretching vibration of O–H groups.^20^ As evident in the spectra, the intensity and width of this band in NaF-based samples, particularly NTi2, are considerably greater than in LiF samples. This increase in intensity and width indicates the presence of a higher amount of surface hydroxyl groups as well as interlayer water and moisture adsorption, likely resulting from increased surface defects, edge degradation, and enhanced MXene surface activation due to the longer etching time in the NaF system. In the region around 2856 and 2961 cm^−1^, a weak band corresponding to C–H and CH_2_ stretching vibration was observed, which is likely attributed to the adsorption of environmental organic species or reaction residues and does not play a decisive role in the main MXene structure.^46^ The band appearing around 1606 cm^−1^, attributed to C=O vibration, showed greater clarity in NaF samples, especially NTi2, and is likely due to CO_2_ adsorption from the environment or the formation of surface carbonyl groups resulting from increased reactivity of MXene sheets and prolonged contact with aqueous medium and air. This is consistent with the increased intensity of the O–H band in these same samples and reflects more severe surface chemistry modification but with lower stability. Distinct bands in the 1011–1253 cm^−1^ region, attributed to Ti–O, C–Ti–O, and Ti–F vibrations, were observed in all MXene samples, but in LiF samples these bands are narrower and more regular, whereas in NaF samples, particularly with increasing etching time, this region has broadened.^47^ This broadening likely indicates a more heterogeneous distribution of functional groups, increased structural disorder, and simultaneous formation of various Ti–O and Ti–F bonds. In the lower frequency region, around 511–806 cm^−1^, bands related to Ti–C and Ti–O vibrations were observed, the presence of which demonstrated that the main Ti–C framework of the MXene is preserved, although the reduced clarity of these bands in the NTi2 sample may be an indication of relative weakening of the structural skeleton due to prolonged etching. Additionally, this band showed higher intensity in the LTi2 sample, which is likely consistent with the peak corresponding to TiO_2_ observed in the XRD analysis.

#### 3.1.4 BET

**Fig. 7:**
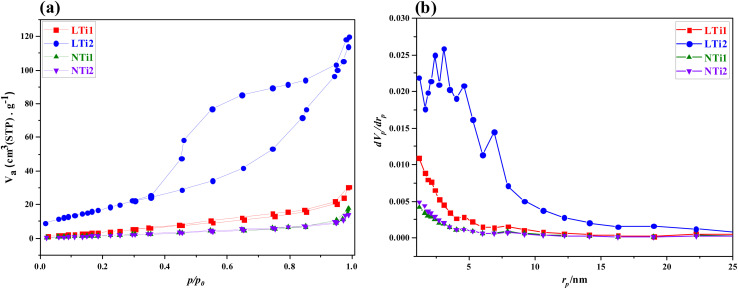
(a) Adsorption–desorption isotherms and (b) BJH model of LTi1, LTi2, NTi1, and NTi2.

The results of adsorption–desorption analysis are presented in Fig. 7a and Table 4. In the LTi1 sample, the lowest degree of structural development was observed, such that calculations using different models yielded a surface area of 19.67 m^2^ g^−1^ and a total pore volume of 0.0468 cm^3^ g^−1^, indicating limited layer separation and retention of a compact multilayer structure, which showed good agreement with SEM and XRD. The Langmuir surface area (≈10 m^2^ g^−1^) was also low, reflecting limited nitrogen access to internal surfaces. The average pore diameter was approximately 9.5 nm, demonstrating that porosity was predominantly of weak mesoporous type arising from surface defects. These results were in complete accordance with FTIR (low OH^−^ intensity) and indicated that etching under these conditions had not yet led to effective delamination. In the LTi2 sample, the best performance in terms of BET parameters was observed. The surface area increased to 73.89 m^2^ g^−1^ and the total pore volume to 0.18 cm^3^ g^−1^, indicating extensive layer separation and creation of abundant diffusion pathways for nitrogen gas.^48^ The high *C* constant value (19.03) demonstrated strong adsorbent–adsorbate interaction and a highly active surface. BJH results also showed a significant increase in mesopore surface area (92.7 m^2^ g^−1^), resulting from interlayer spacing opening and effective delamination (See Fig. 7b). This behavior was in complete agreement with FTIR (high intensity of OH^−^ and Ti–O bands) and XRD (significant shift of the (002) peak), demonstrating that temperature increase in the LiF system was the most effective factor for developing the active surface of MXene. In the NTi1 sample, the surface area was 8.95 m^2^ g^−1^ and the pore volume was 0.027 cm^3^ g^−1^, which was even lower than LTi1. This indicated that at low NaF ratio, the etching process was mild, and although Al removal occurred, limited layer separation remained. The relatively larger average pore diameter (12.1 nm) indicated the formation of open but few mesopores. This behavior was consistent with FTIR (moderate OH^−^ and Ti–O intensity) and XRD (limited structural opening) and reflected the formation of MXene with high structural stability but lower specific surface area. Although the NTi2 sample had a much lower specific surface area (11.11 m^2^ g^−1^) compared to LTi2, its structural characteristics were noteworthy. The decrease in total pore volume (0.021 cm^3^ g^−1^) accompanied by a reduction in average pore diameter (7.73 nm) indicated that the hydrothermal process caused rearrangement and homogenization of the structure. In this case, instead of creating large and irregular pores, the layers transformed into a more stable structure with uniform mesopores, which was in complete agreement with FTIR (high intensity of OH^−^ and interlayer water) and SEM, which demonstrated increased water penetration into the layers and colloidal stability.

Surface area and porosity parameters obtained from BET, Langmuir, *t*-plot, and BJH models for different synthesized products

**Table:** 

Name	BET				
	*V*_m_ [cm^3^(STP) g^−1^]	*a*_s,BET_ [m^2^ g^−1^]	*C*	Total pore volume (*p*/*p*_0_ = 0.990) [cm^3^ g^−1^]	Mean pore diameter [nm]
LTi1	4.5195	19.671	6.9441	0.046841	9.5248
LTi2	16.977	73.89	19.034	0.1802	9.756
NTi1	2.0554	8.946	7.0104	0.027052	12.096
NTi2	2.5531	11.112	3.3889	0.021469	7.7281

**Table:** 

Name	Langmuir plot			*t* plot (adsorption branch)		BJH plot (adsorption branch)		
	*V*_m_ [cm^3^_(STP)_ g^−1^]	*a*_s,Lang_ [m^2^ g^−1^]	*B*	*a*_1_ [m^2^ g^−1^]	*V*_1_ [cm^3^ g^−1^]	*v*_p_ [cm^3^ g^−1^]	*r*_p,peak_ (area) [nm]	*a*_p_ [m^2^ g^−1^]
LTi1	2.2966	9.996	0.3644	5.4794	0	0.051731	1.22	28.265
LTi2	12.834	55.862	1.7515	49.847	0	0.1883	3.1	92.73
NTi1	1.0348	4.504	0.5252	2.8975	0	0.028812	1.22	11.475
NTi2	1.0695	4.6552	0.195	1.7185	0	0.02394	1.22	12.735

### 3.2 Adsorption study

**Fig. 8:**
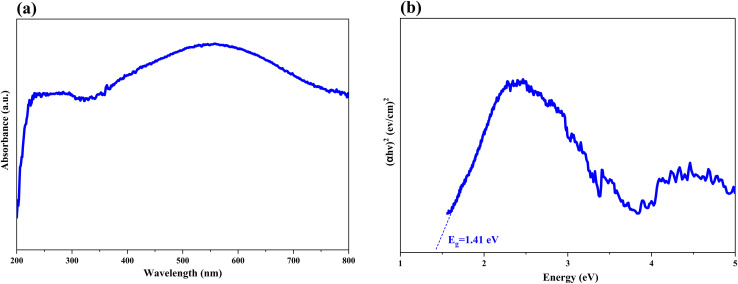
(a) UV-vis absorption spectrum and (b) Tauc plot of LTi2.

Fig. 8a presents the UV-vis absorption spectrum of the LTi2 sample, demonstrating light absorption across a broad wavelength range, which indicates its suitable capability for interacting with light in the ultraviolet and visible regions. The presence of a broad absorption band in the visible region, with a maximum around 550–600 nm, was attributed to the layered structure of MXene and the presence of surface functional groups, which can facilitate electronic transitions in the visible region. In Fig. 8b, using the Tauc plot of (*αhν*)^2^*versus* photon energy, the optical band gap of the sample was determined. Based on the extrapolation of the linear region, the band gap was estimated to be approximately 1.41 eV. This relatively low band-gap value indicated that the LTi2 sample was capable of efficiently absorbing visible light. Therefore, visible-light irradiation was selected for the pollutant-removal experiments to evaluate the potential contribution of light absorption to the overall removal performance in aqueous media.

To better evaluate the role of the remarkable specific surface area of the synthesized MXene and to examine the extent to which this characteristic is observable in the actual performance of the material, the efficiency of the samples in adsorption as well as photocatalytic degradation of several important pollutants was investigated.

**Fig. 9:**
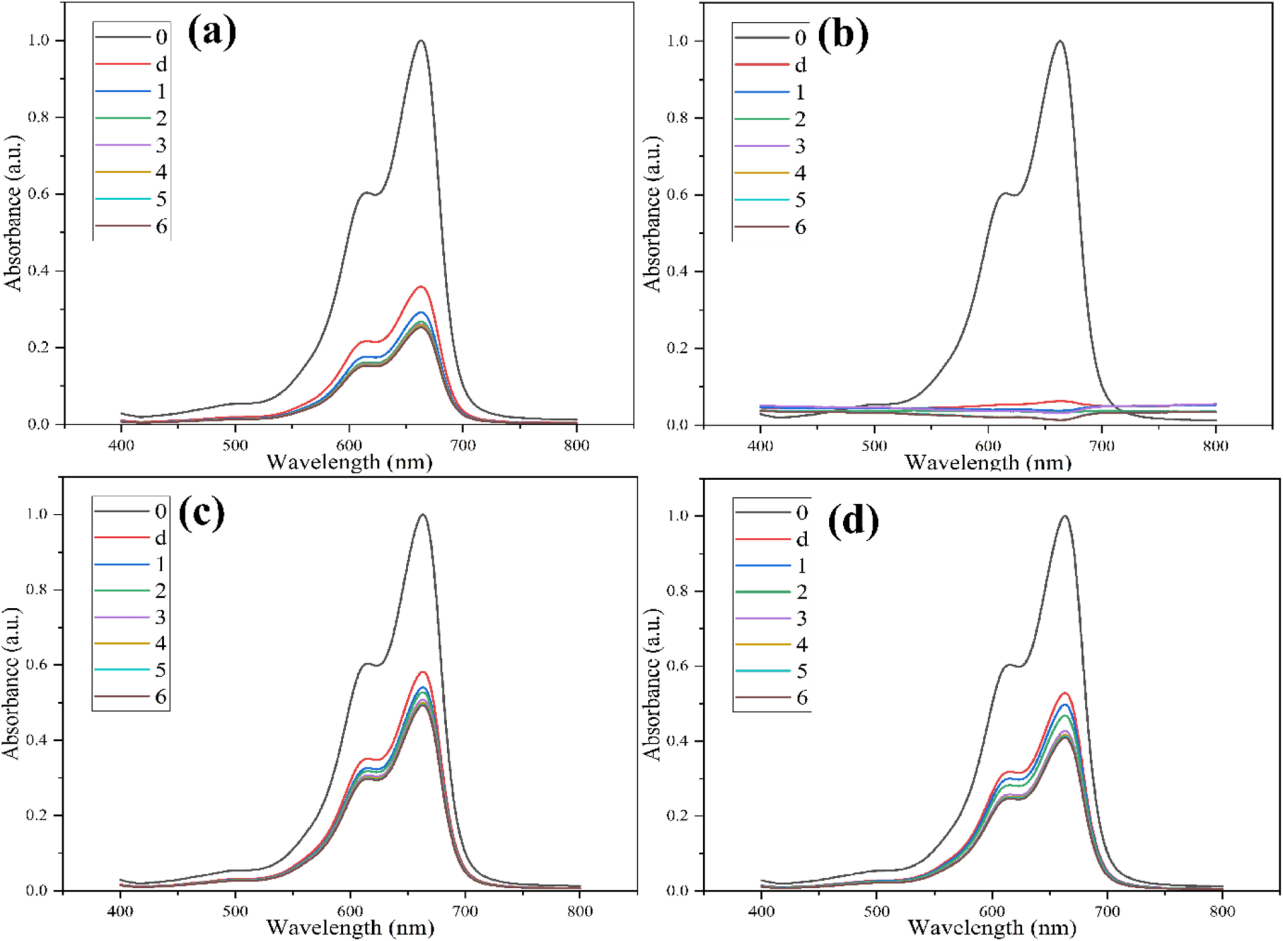
UV-vis absorption spectra of MB (10 ppm) during the photocatalytic degradation process; (a) LTi1, (b) LTi2, (c) NTi1, and (d) NTi2.

The results obtained from the changes in absorption intensity over time are presented in Fig. 9a–d and 13a for all samples. Among the different samples, all showed acceptable capability in removing MB with an initial concentration of 10 ppm; however, the difference in performance between them was clearly observable. The LTi2 sample exhibited a completely distinct performance from other synthesis methods; such that it reduced more than 93% of the target dye concentration within 60 minutes and more than 98% through a complete photocatalytic process (1 hour of darkness and 6 hours of visible light). This superiority is directly related to the structural characteristics of this sample. The higher specific surface area, increased number of accessible pores, and the presence of active surface functional groups all created conditions for dye molecules to approach the surface more easily and anchor onto it.

In Fig. 15b, the color changes of the MB solution with an initial concentration of 10 ppm during the process are shown. Number zero corresponds to the initial solution with blue color, and within the first hour, the solution became colorless; this reduction was also consistent with the numerical results in Table 5.

**Table 5:** Variation of the relative concentration ratio *C*/*C*_0_ over time for the adsorption of different pollutants (MB, TC, CBZ, and PhP) using various synthesized MXene samples (LTi1, LTi2, NTi1, NTi2)

Pollution	Sample type	*C*/*C*_0_						
		*d*	1	2	3	4	5	6
MB 10 ppm	LTi1	0.359	0.293	0.268	0.262	0.259	0.254	0.253
	LTi2	0.064	0.039	0.035	0.033	0.015	0.015	0.014
	NTi1	0.582	0.541	0.528	0.508	0.499	0.495	0.493
	NTi2	0.528	0.498	0.469	0.428	0.418	0.414	0.409
TC 40 ppm	LTi1	0.848	0.816	0.793	0.781	0.776	0.771	0.768
	LTi2	0.707	0.687	0.654	0.609	0.524	0.436	0.385
	NTi1	0.954	0.931	0.914	0.903	0.892	0.883	0.881
	NTi2	0.918	0.903	0.886	0.873	0.864	0.855	0.853
MB 40 ppm	LTi2	0.515	0.479	0.448	0.427	0.415	0.408	0.407
CBZ 40 ppm	LTi2	0.811	0.718	0.694	0.643	0.637	0.631	0.628
PhP 10 ppm	LTi2	0.117	0.097	0.082	0.073	0.068	0.064	0.062

This behavior indicates that the optimal combination of etching conditions and synthesis temperature in the LTi2 sample not only created an appropriate interlayer spacing but also increased the number of active surface sites; sites that, through electrostatic forces, π–π interactions, and physical and chemical surface adsorption, retained MB molecules alongside them. Therefore, the highest observed adsorption efficiency was completely consistent with the measured structural parameters (including BET).

To investigate whether partial oxidation in the LTi2 sample and the potential formation of a TiO_2_ surface phase could play an effective role in the photocatalytic process, two relatively simple and confirmatory experimental methods were employed. In the first step, after conducting the photocatalytic reaction in the visible light chamber, the remaining nanoparticles were separated from the reaction medium, dried, and subsequently subjected to UV-vis DRS analysis.

The results of this test, presented in Fig. 11, demonstrated that throughout the complete photocatalysis process, the amount of residual MB decreased compared to the control sample that was kept only under dark conditions. This slight difference in optical absorption in the region corresponding to MB is likely an indication of a weak photocatalytic degradation process occurring on the oxidized surface of the MXene. In other words, partial oxidation of the MXene surface and its enhanced light absorption, along with the formation of minor TiO_2_ phases, have been able to generate some degree of photocatalytic activity under visible light irradiation, although this effect is so limited that it cannot be considered significant from a practical standpoint.

**Fig. 10:**
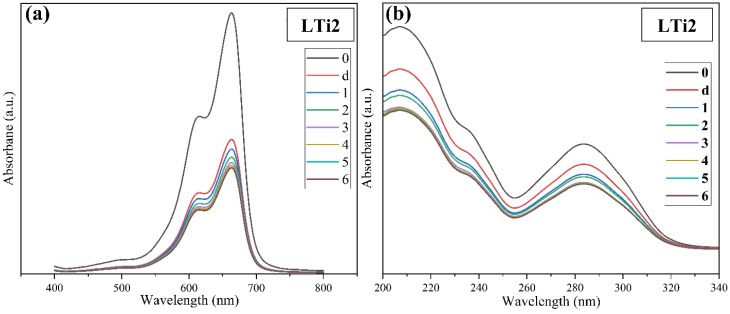
UV-vis absorption spectra of (a) MB and (b) CBZ (40 ppm) during the photocatalytic degradation process at different irradiation times.

To ensure the validity of this conclusion, another experiment was performed using a MB solution with a concentration of 40 ppm to examine the material's behavior at higher concentrations as well (See Fig. 13c). The data obtained from UV-vis spectroscopy in Fig. 10a, 12c, and Table 5 showed that during the dark stage, the dye adsorption process occurred efficiently with high performance, and within 60 minutes, more than 48% of the initial dye was adsorbed. However, after exposure to visible light irradiation for 6 hours, the reduction in optical absorption was only about 11% relative to the initial value from the dark stage. This small decrease suggests that no significant or sustained photocatalytic reaction occurred on the material surface, and actual degradation of dye molecules was not achieved to any significant extent.

**Fig. 11:**
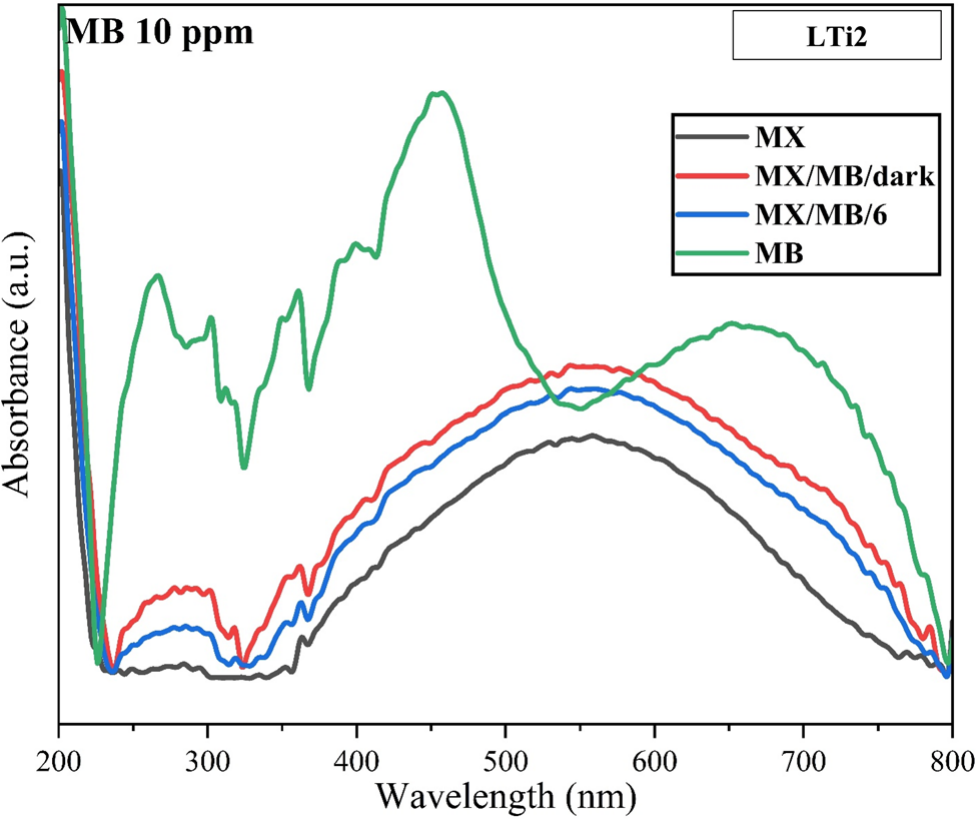
UV-vis DRS reflectance spectra of the LTi2 sample before and after *vis* irradiation during the photocatalytic degradation of MB (10 ppm).

It can be concluded that although partial oxidation has led to the formation of TiO_2_ centers on the MXene surface, the extent of these changes has not been sufficient to generate effective photocatalytic performance, and the primary structure of the MXene still possesses predominantly adsorbent characteristics rather than photocatalytic ones. Furthermore, it can be stated that increasing the density of surface groups and controlled optimization of the oxidation process could contribute to improved photocatalytic performance in the future, but under current conditions, the dominant role of MXene in pollutant removal is limited to their physical and chemical adsorption.

**Fig. 12:**
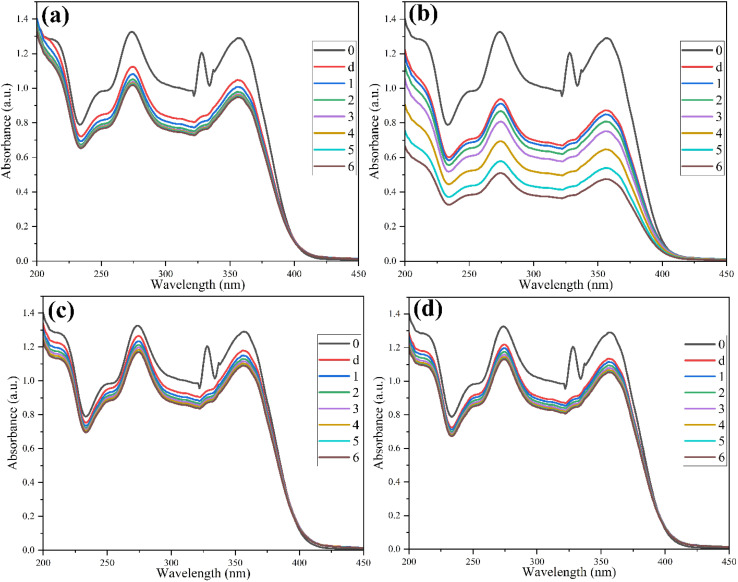
UV-vis absorption spectra of TC (40 ppm) during the photocatalytic degradation process at different irradiation times; (a) LTi1, (b) LTi2, (c) NTi1, and (d) NTi2.

**Fig. 13:**
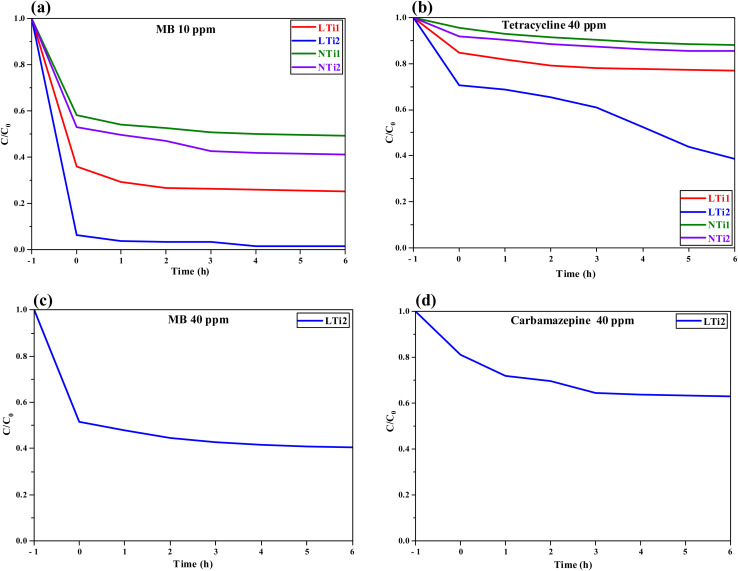
Relative concentration variation *C*/*C*_0_ during the adsorption of different organic pollutants over MXene samples synthesized by various etching conditions. (a) MB (10 ppm), (b) TC (40 ppm), (c) MB (40 ppm) and, (d) CBZ (40 ppm) using LTi2.

Examination of the UV-vis absorption spectra in Fig. 12a to d, 13b, and Table 5 reveals the changes in TC concentration over time in the presence of four different MXene samples. In all graphs, the curve corresponding to time zero represents the initial TC concentration, which is observed with characteristic absorption peaks in the range of approximately 260 to 370 nanometers. The results showed that the LTi2 sample exhibited superior performance compared to other samples in the adsorption and photocatalytic degradation of TC. During the adsorption stage, a noticeable reduction in the intensity of absorption peaks was observed, and more than 30% of the initial drug concentration was removed from the solution. This behavior indicates a type of surface interaction between the active sites of the LTi2 sample and TC molecules. The presence of surface functional groups on the MXene, as well as the layered structure and appropriate interlayer spacing in LTi2, have likely facilitated the penetration of drug molecules and enhanced electrostatic interactions or hydrogen bonding with the material surface.

After exposure to visible light irradiation for 6 hours, the intensity of absorption peaks in the LTi2 sample decreased further, and the overall TC removal reached more than approximately 60%. The continued decline in absorption intensity following the dark stage indicated photodegradation of the drug alongside the LTi2 sample. Although this photocatalytic activity was not particularly strong, it demonstrated that LTi2 is capable of contributing to some extent to the generation of active species such as oxidizing radicals under visible light irradiation and assisting in breaking down the TC molecular structure. This behavior was consistent with the photocatalytic test results reported in other sections of the research but exhibited higher efficiency compared to the others. The numerical results are summarized in Table 5. In contrast, the LTi1, NTi1, and NTi2 samples, similar to other pollutants, showed less reduction in the intensity of TC absorption peaks. In these samples, the main decrease in absorption intensity occurred primarily during the dark stage and was related to the initial adsorption of drug molecules onto the material surface. After the onset of visible light irradiation, no significant change was observed in the spectra, and the curves corresponding to times from 1 to 6 hours followed approximately similar trends.

The UV-vis spectrum shown in Fig. 10b and the *C*/*C*_0_ plot in Fig. 13d illustrate the changes in absorption of CBZ at an initial concentration of 40 ppm in the presence of the LTi2 sample over a time interval from zero to 6 hours. In this sample, a decline in spectral absorption was observable in the initial stages, but it was lower compared to other pollutants, and the absorption intensity decreased slightly over time. This is likely related to the structural nature of CBZ and its weaker interaction with the MXene surface. CBZ is a relatively stable molecule with an aromatic structure and limited water solubility, lacking strong charged groups that could readily establish electrostatic interactions with the functional groups on the MXene surface. Consequently, its adsorption process faces greater limitations compared to cationic dyes such as MB.

The quantitative results summarized in Table 5 also confirmed this trend. The LTi2 sample was able to adsorb approximately 18% of the CBZ present in the solution during the initial adsorption stage within 60 minutes. Subsequently, as the process continued under visible light irradiation, the removal amount gradually increased, reaching approximately 37% after 6 hours. The gradual increase in removal during this stage indicated that, in addition to surface adsorption, the photocatalytic degradation process also played an effective role in reducing the concentration of this drug.

**Fig. 14:**
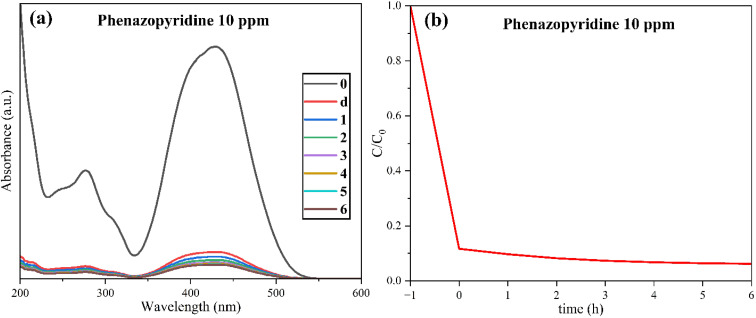
(a) UV-vis absorption spectra of PhP (10 ppm) during the photocatalytic degradation process at different irradiation times and (b) relative concentration variation *C*/*C*_0_ during the adsorption of PhP over LTi2.

Fig. 14a shows the removal trend and the decrease in spectral intensity of PhP at an initial concentration of 10 ppm in the presence of the LTi2 sample. The left plot displays the absorption spectrum of PhP in the range of 200 to 600 nm, where the curve corresponding to time zero exhibited high absorption intensity in specific spectral regions, indicating the presence of the initial concentration of the substance. As contact time progressed (from 1 to 6 hours), the intensity of the absorption peaks decreased significantly. This sharp decline was very pronounced in the first hour and continued with a more gradual decreasing trend in subsequent hours. Such behavior indicated that the adsorption process in the dark by the LTi2 sample initiated very rapidly, and a major portion of the substance was adsorbed within a short time, the values of which are provided in the table. The right plot also showed a rapid decrease in *C*/*C*_0_ in the initial minutes, confirming the high adsorption efficiency of the sample at the onset of the reaction.

**Fig. 15:**
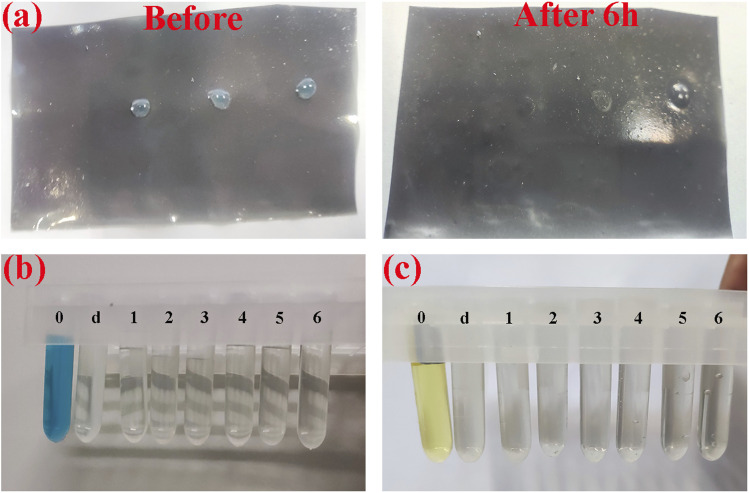
Photographs demonstrating the practical application of the MXene-embedded AcL film for pollutant removal from aqueous solutions: (a) appearance of the film before and after 6 h of the adsorption/photocatalytic process, (b) color change of MB solution at different times, (c) color change of PhP solution at different times, indicating gradual pollutant removal.

Fig. 15c presents a visual representation of the PhP drug solution at a concentration of 10 ppm. At the beginning of the process, the solution had a distinct yellow color, but after 1 hour, the solution became clear. This color change indicated a reduction in PhP concentration due to adsorption and was consistent with the numerical results.

Fig. 15a shows the AcL film containing LTi2 nanoparticles before use and after 6 hours of photocatalytic process. As observed, the film maintained its integrated structure after the experiment, and the nanoparticles remained within the polymeric matrix. Additionally, after the dye adsorption process on the film surface, the blue water droplet became colorless. This demonstrated that immobilization of the optimized LTi2 sample in an AcL film enabled practical application of this material in the purification of organic pollutants without noticeable reduction in efficiency. Furthermore, the film structure facilitated catalyst separation from the solution and enhanced its recoverability and reusability in water treatment processes.

## 4 Conclusion

In this study, Ti_3_C_2_T_*x*_ was synthesized from the parent Ti_3_AlC_2_ phase through selective etching and subsequently employed with the aim of optimizing their performance as adsorbents and photocatalysts for the removal of organic pollutants from water. By varying the etching agent type (LiF/HCl compared to NaF/HCl) as well as adjusting the reaction temperature and duration, four MXene samples with different layered structures, specific surface areas, and surface chemistries were prepared, and their performance in removing MB dye and pharmaceutical compounds TC, CBZ, and PhP was evaluated.

Among the synthesized samples, LTi2 exhibited superior performance. The more severe etching conditions in this synthesis route resulted in a more delaminated structure, higher specific surface area, and wider interlayer spacing accompanied by appropriate functional groups on the surface. These characteristics directly led to the high efficiency of this sample in pollutant adsorption; such that at 10 ppm concentration, it removed over 93% of MB and 88% of PhP within 60 minutes. At the higher concentration of 40 ppm, this sample was able to remove 48%, 29%, and 18% of MB, TC, and CBZ, respectively, indicating its stronger structural compatibility with MB and PhP compared to the more resistant drugs TC and CBZ.

In addition to its outstanding adsorption capability, LTi2 was the only sample that exhibited a noticeable enhancement in pollutant removal under visible-light irradiation. This sample, after the dark adsorption stage, achieved removal of over 98% for MB and over 93% for PhP at 10 ppm concentration under visible light irradiation, and at 40 ppm concentration also provided considerable pollutant reduction; such that approximately 59–64% total removal was achieved for MB and TC, and about 37% for CBZ. In contrast, samples LTi1, NTi1, and NTi2 showed virtually no additional photocatalytic activity, and their performance was essentially limited to initial adsorption; this clear difference demonstrated the determining role of synthesis conditions and surface groups in creating a bifunctional MXene. To prevent nanoparticle release and secondary pollution, the optimized LTi2 sample was immobilized in an AcL polymeric matrix. This composite film maintained its structural stability in contact with contaminated solutions and enabled complete removal of MB (10 ppm), while also making easy recovery and system reuse possible. It can be stated that intelligent adjustment of etching conditions and synthesis parameters is an effective tool for engineering MXene structure and surface chemistry and enhancing its capability in environmental applications. In this context, the LTi2 sample was demonstrated to be an efficient adsorbent for the visible-light-assisted removal of dyes and pharmaceutical compounds from water, highlighting the potential of structure-engineered MXenes for advanced water treatment applications.

## Conflicts of interest

The authors declare that they have no known competing financial interests or personal relationships that could have appeared to influence the work reported in this paper.

## Data Availability

All data that support the findings of this study are included with the article.

## References

[cit1] Zhussupbekov K. (2025). *et al.*, Self-Assembled Moiré Superlattices of Ti3C2Tx MXene for Future Twistronic Applications (Adv. Sci. 45/2025). Adv. Sci..

[cit2] Naguib M. (2014). *et al.*, 25th anniversary article: MXenes: a new family of two-dimensional materials. Adv. Mater..

[cit3] Ghamarpoor R., Ramezanzadeh B. (2026). A Multifunctional Hybrid MXene/ZIF/Semiconductor-Based Coating for Photocatalytic Degradation and Long-Term Marine Corrosion Protection. Adv. Ind. Eng. Polym. Res..

[cit4] Ghamarpoor R., Ramezanzadeh B. (2026). Epoxy coatings reinforced with V2C-MXene nano-reservoirs decorated by horned BZIF structures for advanced self-healing and corrosion protection. Prog. Org. Coat..

[cit5] Ye H. (2025). *et al.*, Advanced MXene/graphene oxide/lignosulfonate inks for 3D printing thick electrodes with vertically aligned pores to dually boost mass loading and areal capacitance. Adv. Funct. Mater..

[cit6] Zhou Q. (2025). *et al.*, Interfacial shear strength of MXene interfaces. Cell Rep. Phys. Sci..

[cit7] Zhou Y. (2025). *et al.*, Additive-Free Ti3C2Tx MXene/Carbon Nanotube Aqueous Inks Enable Energy Density Enriched 3D-Printed Flexible Micro-Supercapacitors for Modular Self-Powered Systems. Carbon Energy.

[cit8] Zhou J. (2022). *et al.*, Maximizing ion permselectivity in MXene/MOF nanofluidic membranes for high-efficient blue energy generation. Adv. Funct. Mater..

[cit9] Ran L. (2023). *et al.*, Liquid metal assisted fabrication of MXene-based films: Toward superior electromagnetic interference shielding and thermal management. J. Colloid Interface Sci..

[cit10] Alahvirdi F. (2026). *et al.*, 2D-vanadium carbide MXene: Influence of synthesis methods, etching agents, reaction time, and temperature on structural properties. Mater. Sci. Eng., B.

[cit11] Prakash N. J., Kandasubramanian B. (2021). Nanocomposites of MXene for industrial applications. J. Alloys Compd..

[cit12] Peng J. (2019). *et al.*, Surface and heterointerface engineering of 2D MXenes and their nanocomposites: insights into electro-and photocatalysis. Chem.

[cit13] Parajuli D. (2022). *et al.*, Advancements in MXene-polymer nanocomposites in energy storage and biomedical applications. Polymers.

[cit14] Wang Y., Wang J., Wen Q. (2021). MXene/graphene oxide heterojunction as a saturable absorber for passively q-switched solid-state pulse lasers. Nanomaterials.

[cit15] Ghamarpoor R., Ramezanzadeh B. (2026). Rational design of a multifunctional bimetallic ZIFs-V2C MXene nanohybrid for enhanced mechanical durability and EMI shielding in polymeric coatings. J. Taiwan Inst. Chem. Eng..

[cit16] Zhang T. (2017). *et al.*, Synthesis of two-dimensional Ti3C2Tx MXene using HCl+ LiF etchant: enhanced exfoliation and delamination. J. Alloys Compd..

[cit17] Xu H. (2026). *et al.*, Enhanced electromagnetic wave absorption through in-situ mineralization constructed robust Fe3O4/PDA/Ti3C2Tx MXene composites with brick-and-mortar microstructure. Ceram. Int..

[cit18] Sokol M. (2019). *et al.*, On the chemical diversity of the MAX phases. Trends Chem..

[cit19] Jawaid A. (2021). *et al.*, Halogen etch of Ti3AlC2 MAX phase for MXene fabrication. ACS Nano.

[cit20] Shuck C. E., Ventura-Martinez K., Goad A., Uzun S., Shekhirev M., Gogotsi Y. (2021). Safe Synthesis of MAX and MXene: Guidelines to Reduce Risk during Synthesis. ACS Chem. Health Saf..

[cit21] Horak P. (2019). *et al.*, Ion Beam Sputtering for Controlled Synthesis of Thin MAX (MXene) Phases. Microsc. Microanal..

[cit22] Natu V. (2020). *et al.*, 2D Ti3C2Tz MXene synthesized by water-free etching of Ti3AlC2 in polar organic solvents. Chem.

[cit23] Kubitza N. (2023). *et al.*, Extending the Chemistry of Layered Solids and Nanosheets: Chemistry and Structure of MAX Phases, MAB Phases and MXenes. ChemPlusChem.

[cit24] Seok S.-H. (2021). *et al.*, Synthesis of high quality 2D carbide MXene flakes using a highly purified MAX precursor for ink applications. Nanoscale Adv..

[cit25] Zheng L. (2010). *et al.*, Ti0. 5Nb0. 5 5AlC4: a new-layered compound belonging to MAX phases. J. Am. Ceram. Soc..

[cit26] Deysher G. (2019). *et al.*, Synthesis of Mo4VAlC4 MAX phase and two-dimensional Mo4VC4 MXene with five atomic layers of transition metals. ACS Nano.

[cit27] Dinesh R., Dinesh S., Siti N. (2023). The effect of fluoride based salt etching in the synthesis of MXene. Mater. Res. Found..

[cit28] Liew J. (2025). *et al.*, A comprehensive characterisation of titanium carbide (Ti3C2) MXene with hydrothermal-assisted in-situ hydrofluoric acid etching. Mater. Sci. Eng., B.

[cit29] Lokhande P. (2025). *et al.*, Etching duration as a key parameter for tailoring Ti3C2Tx MXene electrochemical properties. J. Phys. Chem. Solids.

[cit30] Rajavel K. (2018). *et al.*, Condition optimization for exfoliation of two dimensional titanium carbide (Ti3C2T x). Nanotechnology.

[cit31] Yadav M. (2025). *et al.*, Tailoring Etching Conditions to Unlock the Electrochemical Potential of 2D Ti3C2TX MXene: Yadav, Kumar, Tomar, Singh, and Sharma. JOM.

[cit32] Abdullah N. (2024). *et al.*, Investigating the impact of various etching agents on Ti3C2Tx MXene synthesis for electrochemical energy conversion. FlatChem.

[cit33] Yaman P., Kucukyildirim B. O. (2026). Tailoring Ti3C2Tx MXene multilayers for biomaterial integration: synthesis, characterization, and cytotoxicity. Mater. Sci. Eng., B.

[cit34] Yilmaz T. (2025). *et al.*, One-Step Synthesis of Metastable Mo2AlB2 from MoAlB Using Gaseous HCl. Inorg. Chem..

[cit35] Cockreham C. B. (2022). *et al.*, Energetic stability and interfacial Complexity of Ti3C2T x MXenes synthesized with HF/HCl and CoF2/HCl as etching agents. ACS Appl. Mater. Interfaces.

[cit36] Ahmadi B. (2026). *et al.*, Advancements in MBenes: synthesis, Surface chemistry, and photocatalytic application in water pollutants degradation. Adv. Compos. Hybrid Mater..

[cit37] Ghamarpoor R. (2026). *et al.*, Single-step covalent functionalization of CeO2/MXene nanohybrids for high-performance smart photocatalytic nano-coatings. Surf. Interfaces.

[cit38] Ahmadi B. (2025). *et al.*, Methylene blue beyond the dye: A critical review on its role as a benchmark pollutant in photocatalyst design. Results Chem..

[cit39] Tran N. My, Qui Thanh Hoai T., Sreedhar A., Noh J.-S. (2021). Ti3C2Tx MXene playing as a strong methylene blue adsorbent in wastewater. Appl. Surf. Sci..

[cit40] Neshastehgar M., Jamshidi M., Ghamarpoor R. (2025). Self-assembly TiO2@ Silane@ SiO2 core-shell as s-scheme heterojunction photocatalyst against methylene blue degradation: synthesis and mechanism insights. J. Mol. Struct..

[cit41] Pang S. Y. (2025). *et al.*, Fluoride-Free Molten Salt Hydrate-Assisted Synthesis of MXene in Air Down to 150°C. Adv. Funct. Mater..

[cit42] Kumar S. (2020). *et al.*, Effect of Ti 3 C 2 T x MXenes etched at elevated temperatures using concentrated acid on binder-free supercapacitors. RSC Adv..

[cit43] Hantanasirisakul K., Gogotsi Y. (2018). Electronic and optical properties of 2D transition metal carbides and nitrides (MXenes). Adv. Mater..

[cit44] Halim J. (2016). *et al.*, X-ray photoelectron spectroscopy of select multi-layered transition metal carbides (MXenes). Appl. Surf. Sci..

[cit45] Ghidiu M. (2016). *et al.*, Ion-exchange and cation solvation reactions in Ti3C2 MXene. Chem. Mater..

[cit46] Levitt A. (2020). *et al.*, MXene-based fibers, yarns, and fabrics for wearable energy storage devices. Adv. Funct. Mater..

[cit47] Wozniak J., Jastrzębska A., Olszyna A. (2022). Challenges and opportunities in tailoring MAX phases as a starting materials for MXenes development. Mater. Technol..

[cit48] Alhabeb M. (2017). *et al.*, Guidelines for synthesis and processing of two-dimensional titanium carbide (Ti3C2T x MXene). Chem. Mater..

